# Clinical findings and assessment of factors associated with survival in dogs presenting with hyperbilirubinaemia: 115 cases in Victoria, Australia (2015–2020)

**DOI:** 10.1002/vro2.42

**Published:** 2022-08-16

**Authors:** Abigail Brough, Charles Caraguel, Susan Ciaravolo, Alison Stickney

**Affiliations:** ^1^ Peninsula Vet Emergency and Referral Hospital Mornington Victoria Australia; ^2^ University of Adelaide Adelaide South Australia Australia

## Abstract

**Introduction:**

Hyperbilirubinaemia is an important clinicopathological finding in canine medicine. The objectives of this study were to describe the clinical presentation and outcome of dogs with hyperbilirubinaemia; also to identify factors associated with survival.

**Materials and methods:**

Retrospective study of dogs with hyperbilirubinaemia from two referral centres in South Australia (2015–2020). Signalment, clinical signs, clinicopathological data, diagnosis and outcome were obtained from searching clinical records. Univariable analysis and logistic regression modelling were used to compare outcomes and overall survival.

**Results:**

A total of 115 cases were included. The most common clinical signs were vomiting (63.5%), anorexia (62.6%), lethargy (55.7%) and pyrexia (18.3%). Pre‐hepatic icterus was diagnosed in 18 cases (15.7%), hepatic icterus in 51 cases (44.3%) and post‐hepatic icterus in 42 cases (36.5%). The median survival time across all cases was 40 days (95% confidence interval [CI]: 9–126 days). There was an increased risk of death in dogs with serum bilirubin greater than 60 μmol/L at diagnosis (odds ratio [OR] = 3.55; 95% CI: 1.53–8.22; *p*‐value = 0.003) and in dogs with pre‐hepatic icterus compared to hepatic (OR = 4.35; 95% CI: 1.18–16.0; *p*‐value = 0.027) and post‐hepatic icterus (OR = 6.52; 95% CI: 1.67–25.5; *p*‐value = 0.007).

**Conclusions:**

Pre‐hepatic icterus was associated with a significantly higher risk of death than hepatic and post‐hepatic icterus. Serum bilirubin >60 μmol/L at diagnosis was associated with a significantly shorter median survival time. This cut‐off may be useful in discussions with owners regarding pursuing further diagnostic investigation and treatment. Further prospective studies are needed to prove the validity of this cut‐off.

## INTRODUCTION

Hyperbilirubinaemia is the accumulation of excess bilirubin in the blood stream and occurs due to failure of normal bilirubin metabolism. Bilirubin is formed from the breakdown of haem pigment and is the end product of the breakdown of red blood cells by the mononuclear phagocytic system. Unconjugated bilirubin is water insoluble, so it is transported to the liver bound to albumin, whereupon it is taken up by hepatocytes and undergoes conjugation via glucuronidation. Bilirubin glucuronides then undergo active transport into bile canaliculi before storage in the gall bladder and ultimately excretion, primarily in faeces.^1^ Hyperbilirubinaemia leads to the accumulation of bilirubin pigment in the blood and tissues (icterus) and can be classified as pre‐hepatic, hepatic or post‐hepatic.^1^

Clinically apparent icterus (including yellowing of the skin, sclera and mucous membranes) is commonly recognised at a bilirubin concentration greater than 35 μmol/L.^1^ Pre‐hepatic icterus occurs when the breakdown of red blood cells in circulation exceeds the conjugation capacity of the liver, resulting in excess levels of unconjugated bilirubin. Examples of diseases resulting in pre‐hepatic icterus in dogs include immune‐mediated haemolytic anaemia (IMHA), infection with haemoparasites such as *Babesia canis* and haemolysis of incompatible blood transfusions. Hepatic icterus occurs when there is reduced hepatocyte function or intrahepatic cholestasis, resulting in the accumulation of conjugated and unconjugated bilirubin. Examples of hepatic icterus in dogs include infectious hepatitis/cholangiohepatitis, hepatic neoplasia, toxic hepatopathies and canine chronic hepatitis. Post‐hepatic icterus occurs secondary to obstruction of the biliary outflow, with excess conjugated bilirubin entering the circulation. This can occur with conditions such as gall bladder mucocoeles (GBM) and extra‐hepatic bile duct obstruction (EHBDO) from either extra‐mural (pancreatitis, pancreatic masses, right cranial quadrant abdominal masses) or intra‐mural (cholelithiasis, biliary tract neoplasia) causes. Measurement of conjugated and unconjugated bilirubin as a means of determining the icterus type is of minimal clinical use due to well‐recognised overlap between the forms.^2^

The presence of hyperbilirubinaemia has previously been reported to be a negative prognostic factor in certain diseases, such as GBM,^3^, ^4^ dogs undergoing gall bladder surgery,^5^ IMHA^6^, ^7^, ^8^, ^9^, ^10^ and acute or chronic hepatitis^11^, ^12^; however, few studies have assessed the magnitude of hyperbilirubinaemia as a prognostic factor. One study assessed a number of prognostic factors associated with survival in dogs undergoing exploratory celiotomy and found a pre‐operative bilirubin concentration of 40 μmol/L to be significantly associated with non‐survival (compared to a pre‐operative concentration of 5.5 μmol/L for survivors).^13^ The objectives of this study were (1) to describe a population of dogs presenting with hyperbilirubinaemia and (2) to determine whether serum bilirubin concentration at presentation could be predictive of underlying pathology and overall survival.

## METHODS

### Case selection

This was a retrospective study performed across two veterinary hospitals in southeastern, suburban Melbourne, Australia. Cases were acquired through both the primary emergency and critical care departments and through the respective referral medical or surgical services. All dogs were client owned; all investigations, interventions and treatments were performed at the attending clinician's discretion. Electronic medical records between 2015 and 2020 were searched for the following terms: hyperbilirubinaemia, jaundice and icterus. The minimum database for inclusion was complete blood count and full serum biochemistry—to include serum bilirubin concentration exceeding the upper reference laboratory limit of 4 μmol/L.

### Medical records review

Information extracted from the medical records included patient age, gender, neuter status, Australian kennel club breed category, clinical signs (presence or absence of lethargy, anorexia, vomiting, diarrhoea, abdominal pain or pyrexia—defined as a rectal temperature greater than 39.2°C), serum bilirubin concentration at initial presentation, final diagnosis (where available), hospitalisation time and survival. Follow‐up data were obtained through primary care veterinarians or direct contact with the owner if it was not available from searching the medical records. Where direct contact with owners was needed, verbal consent was obtained to include the patient data in the study. Cases were de‐identified (by removal of patient name and surname) and instead assigned a reference number for data storage in a spreadsheet format in Microsoft Excel.

Each case was assigned a classification of pre‐hepatic, hepatic or post‐hepatic icterus after review of the medical records. Criteria for pre‐hepatic icterus were determined in line with published guidelines for IMHA and included demonstration of intravascular or extravascular haemolysis via the presence of haematocrit <0.35 L/L or packed cell volume <35%, in addition to one or more of the following: haemolysed serum, haemoglobinuria, spherocytosis, positive saline agglutination test or a positive Coomb's test.^14^ Pre‐hepatic icterus was also assigned if there was documented evidence of post‐transfusion haemolysis. Post‐hepatic icterus was assigned based on demonstration of a GBM or intraluminal or extraluminal obstruction of the common bile duct (based on imaging or surgical findings of bile duct distension). Classification of hepatic icterus was based on the diagnosis of liver pathology via cytological or histopathological evaluation. In cases where this information was not available, hepatic icterus was assumed in cases where there was biochemical evidence of hepatocellular damage and pre‐hepatic and post‐hepatic causes had been reasonably excluded. Cases were excluded from further analysis if there were insufficient data to determine the type of icterus.

Case outcome was determined via a search of the medical records or via direct telephone communication with the owners or the general practitioner. Cases were followed until death—either due to euthanasia or natural causes—or until the last visit pertaining to the presenting condition in recovered cases. Death was reported as either ‘icterus‐related mortality’ if death or euthanasia was deemed to be directly related to the condition causing hyperbilirubinaemia; or as ‘unrelated’ to the condition if they died for other reasons.

### Statistical analysis

Demographics and clinical presentation of cases were reported as counts and percentages for categorical variables and as medians and ranges for continuous variables. Potential differences between referral hospitals were assessed using an exact Fisher test for categorical variables and a Kruskal‒Wallis equality‐of‐populations rank test for continuous variables.

An assessment of prognosis was made by evaluating the following factors: (i) incidence risk (probability of icterus‐related mortality regardless of time), (ii) incidence rate (probability of icterus‐related mortality over the study period) and (iii) median survival time (MST) (time from diagnosis to icterus‐related mortality).

The overall incidence risk was reported with its binomial exact 95% confidence interval (CI). Univariable analyses were conducted using simple logistic regression models. After selecting among highly collinear variables, only those with unconditional *p*‐values < 0.1 were considered in the final multifactorial logistic regression model. After exploring for potential first‐degree interactions among the selected variables using likelihood ratio tests, the final model was built using a stepwise regression approach.

The overall MST and its 95% CI were obtained by conventional survival analysis based on the non‐parametric Kaplan‒Meier estimation of the survival function. The follow‐up period started at the time of diagnosis and terminated at the time of death due to the condition (icterus‐related mortality) or when the case died for unrelated reasons or when it was seen last (censored).

Case survival was compared using either a non‐parametric log‐rank test (categorical variables only) comparing MST or a semi‐parametric Cox proportional hazard model comparing hazard (risk of death at a given point in time) profiles.

The continuous variables (age at diagnosis and serum bilirubin concentration at presentation) were further analysed by exploring the threshold value at which the hazard ratio was the largest or most significant. This enabled us to establish a statistically significant cut‐off value for serum bilirubin concentration. Variables significant or marginally significant (*p*‐values <0.1) were considered further for multifactorial Cox model building, including exploring potential first‐degree interactions. As only one prognostic factor remained in the final model, we preferred to compare the survival functions for this factor using the non‐parametric log‐rank test and report the respective Kaplan‒Meier survival curves.

*p*‐Values were interpreted at the 5% level of significance (*α* or type‐I error). All analyses were conducted using the statistical package Stata v16.1 (StataCorp, College Station, TX, USA).

## RESULTS

### Case profiles

An initial search of the medical records yielded 132 cases, with 17 cases excluded due to insufficient data. Clinical records from a total of 115 canine cases of hyperbilirubinaemia were included from both referral hospitals (53 from hospital A and 62 from hospital B) between 2015 and 2020. A summary of the demographic data and clinical presentations of those cases is presented in Table 1. The majority of the cases were purebred dogs (73.0%), with 46 different breeds represented. The most commonly reported breeds included the Labrador Retriever (*n* = 12), Border Collie (*n* = 7) and Kelpie (*n* = 4). The median age at presentation was 9 years (range: 10 months–16 years).

**TABLE 1:** Overall and hospital‐specific summaries of demographic data and clinical presentation of 115 canine icterus cases sourced from two Australian veterinary hospitals between 2015 and 2020

	Hospital A		Hospital B		Overall	
Parameters	Count	%	Count	%	Count	%
**Demographics**						
Entire female	1	1.8%	1	1.6%	2	1.7%
Spayed female	26	49.1%	42	67.7%	68	59.1%
Entire male	0	0.0%	2	3.2%	2	1.7%
Neutered male	26	49.1%	17	27.4%	43	37.4%
Age in years at diagnosis, median (range)	53	10 (1–16)	62	9 (1–15)	115	9 (1–16)
**Purebreeds and Australian National Kennel Council breed group** ^22^	40	75.5%	44	71.0%	84	73.0%
Gundog	14	35.0%	8	18.2%	22	26.2%
Hound	1	2.5%	1	2.3%	2	2.4%
Non‐sporting	2	5.0%	2	4.5%	4	4.8%
Terrier	7	17.5%	7	15.9%	14	16.7%
Toy	3	7.5%	10	22.7%	13	15.5%
Utility	6	15.0%	6	13.6%	12	14.3%
Working dog	7	17.5%	10	22.7%	17	20.2%
Crossbreeds	13	24.5%	18	29.0%	31	27.0%
**Clinical presentation**						
Anorexia	35	66.0%	38	61.3%	73	63.5%
Vomiting	40	75.5%	32	51.6%	72	62.6%
Lethargy	28	52.8%	36	58.1%	64	55.7%
Pyrexia	12	22.6%	9	14.5%	21	18.3%
Abdominal pain	12	22.6%	4	6.5%	16	13.9%
Diarrhoea	5	9.4%	3	4.8%	8	7.0%
Serum bilirubin concentration at presentation (μmol/L), median (range)	53	50 (6–282)	62	50.5 (10–320)	115	50 (6–320)
**Icterus type**						
Pre‐hepatic icterus	4	7.5%	14	22.6%	18	15.7%
Hepatic icterus	21	39.6%	30	48.4%	51	44.3%
Post‐hepatic icterus	26	49.1%	16	25.8%	42	36.5%
Missing data	2	3.8%	2	3.2%	4	3.5%

Approximately two‐third of the cases presented with anorexia and vomiting, while just over half presented with lethargy. Approximately one‐fifth of dogs were pyrexic on presentation; only a small minority had abdominal pain or diarrhoea. When broken down by icterus category, the most common presenting clinical sign for cases with pre‐hepatic icterus was lethargy (83% of cases), while for hepatic and post‐hepatic icterus it was vomiting and anorexia/hyporexia (60% and 66%; 85% and 66%, respectively). The median serum bilirubin concentration at presentation was 50 μmol/L (range: 6–320 μmol/L).

The most common type of icterus was hepatic (44.3%), followed by post‐hepatic (36.5%) and pre‐hepatic (15.7%). Of the 115 dogs, 77 (66.9%) underwent abdominal ultrasound examination, 12 (10.4%) had an abdominal CT scan, 25 (21.7%) had liver biopsy samples collected for histopathology, 13 (11.3%) had liver fine needle aspiration of the liver with cytological analysis and 16 (13.9%) had either liver tissue or bile culture and sensitivity.

The icterus type could not be determined for four cases, and these were excluded from further analysis. The first of these cases presented with haemorrhagic gastroenteritis, had marginal elevation of serum bilirubin (12 μmol/L) and was discharged after 1 day of hospitalisation following no further investigation and was alive at the time of writing. The second presented with diarrhoea, lethargy and vomiting and had minor increase of alanine aminotransferase and alkaline phosphatase, with moderate elevation of serum bilirubin (48 μmol/L). No further investigation was undertaken; the dog was discharged 1 day after hospitalisation and was alive at the time of writing. The remaining two cases both presented for grand mal seizures and had marginal elevation of serum bilirubin (14 and 18 μmol/L, respectively). Neither dog underwent further diagnostic investigation; they were discharged following one day of hospitalisation and were lost to follow‐up.

Cases from hospitals A and B were similar, except that hospital B had more female dogs presenting with icterus (*p*‐value = 0.056) and more cases presenting with vomiting (*p*‐value = 0.012) and abdominal pain (*p*‐value = 0.015). Cases from hospital A were more likely to present with post‐ versus pre‐hepatic icterus (*p*‐value = 0.013), whereas there was no significant difference in the incidence of post‐ versus pre‐hepatic icterus in cases from hospital B (Table 1).

The accessed number of cases (*n* = 115) provided 80% study power (type‐II error = 20%) to detect significantly (type‐I error = 5%) a minimum increase of 70.7% (log‐rank test) or 68.6% (Cox model) in the hazard of icterus‐related mortality for any given binary prognostic factor equally distributed among cases (50%/50%).

### Case prognosis

Over the follow‐up period, 57 icteric dogs (49.6%; 95% CI: 40.1–59.0) died or were euthanased due to their condition, five (4.4%) died for unrelated reasons; the remainder of the cases (*n* = 53, 46.1%) were still alive at the end of the follow‐up period.

At the time of writing, 57 out of 115 dogs (49.5%) had died or been euthanased as a result of their condition, with 41 of 115 (35%) failing to survive to discharge. When broken down by icterus category, nine out of 18 (50%) with pre‐hepatic icterus, 16 out of 51 (31%) with hepatic icterus and 16 out of 42 (38%) with post‐hepatic icterus did not survive to discharge.

Table 2 summarises the potential prognostic factors. Please note that these are unconditional associations; therefore, reported odds ratios (ORs) and *p*‐values are different from the conditional associations reported after examination of the final logistic regression model. Pyrexia, serum bilirubin concentration and the icterus type were considered for the final logistic regression construction and remained in the final model with no significant interaction.

**TABLE 2:** Unconditional association of potential demographic and clinical prognostic factors with the risk (logistic regression), survival (log‐rank test) or hazard profile (Cox proportional hazard model) of jaundice‐related death in 115 canine jaundice cases sourced from two Australian veterinary hospitals between 2015 and 2020

		Log‐rank test		Cox proportional hazard model	
Parameters (reference category)	Logistic regression Odds ratio	*p*‐Value	*p*‐Value	Hazard ratio	*p*‐Value
**Demographics**					
Male (female)	0.957	0.907	0.673	0.895	0.684
Desexed (entire)	0.982	0.986	0.555	1.515	0.567
Crossbreed (pure breed)	1.336	0.493	0.548	1.183	0.562
Breed group					
Gundog	1.000	–	0.157	1.000	–
Hound	0.545	0.639	–	0.561	0.581
Non‐sporting	0.655	0.614	–	0.747	0.655
Terrier	0.694	0.568	–	0.783	0.614
Toy	1.636	0.379	–	1.682	0.179
Utility	2.727	0.166	–	1.681	0.237
Working dog	0.636	0.475	–	0.665	0.399
**Clinical presentation**					
Age at diagnosis—continuous scale	1.065	0.251	–	1.049	0.231
>12 years old at diagnosis (≤12 years)	1.773	0.229	0.112	1.598	0.129
Anorexia/hyporexia (not reported or present)	0.619	0.219	0.437	0.818	0.453
Vomiting (not reported or present)	0.576	0.157	0.327	0.776	0.345
Lethargy (not reported or present)	1.591	0.219	0.568	1.163	0.582
Pyrexia (not reported or present)	**0.337**	**0.039**	**0.037**	**0.427**	**0.049**
Abdominal pain^a^ (not reported or present)	0.762	0.617	0.998	1.001	0.998
Diarrhoea^b^ (not reported or present)	0.589	0.483	0.455	0.652	0.473
Serum bilirubin concentration at diagnosis—continuous scale	1.005	0.062	–	1.002	0.154
Serum bilirubin concentration >60 μmol/L at diagnosis (≤60 μmol/L)	**2.822**	**0.007**	**0.010**	**1.936**	**0.014**
**Icterus type**					
Pre‐hepatic	1.000	–	0.131	1.000	–
Hepatic icterus	**0.275**	**0.041**	–	0.572	0.096
Post‐hepatic icterus	**0.214**	**0.017**	–	0.533	0.079
**Others**					
Hospital B (hospital A)	1.375	0.396	0.7375	1.091	0.746

^a^ Abdominal pain was assessed based on recorded physical examination findings by the attending veterinary clinician.

^b^ Diarrhoea was based on history taking from owners; faecal grading was not able to be assigned.

The values in bold are the statistically significant variables, with *p*‐values less than 0.05.

**FIGURE 1:**
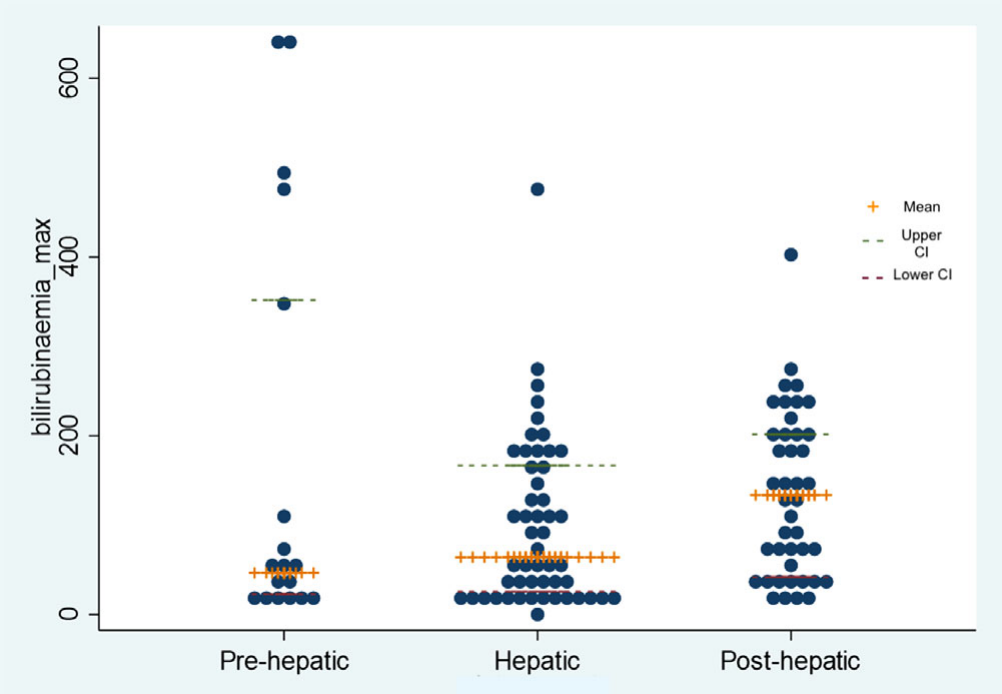
Comparison of the mean and 95% confidence interval (CI) distribution of serum bilirubin at diagnosis (mmol/L) against the icterus category.

A serum bilirubin concentration greater than 60 μmol/L at presentation was significantly associated with an increased risk of icterus‐related mortality (OR = 3.55; 95% CI: 1.53–8.22; *p*‐value = 0.003). Dogs with pre‐hepatic icterus were also significantly more likely to die than dogs with hepatic or post‐hepatic icterus, as demonstrated in Figure 1 (OR = 4.35; 95% CI: 1.18–16.0; *p*‐value = 0.027; OR = 6.52; 95% CI: 1.67–25.5; *p*‐value = 0.007, respectively).

**FIGURE 2:**
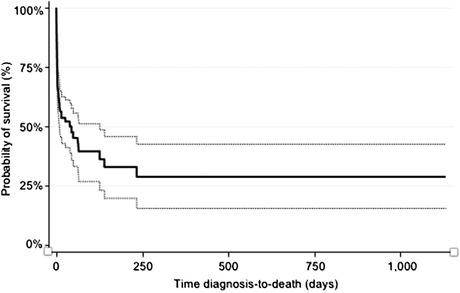
Kaplan‒Meier survival curve and its 95% confidence interval (dashed lines) across a series of 115 canine icterus cases sourced from two Australian veterinary hospitals between 2015 and 2020.

**FIGURE 3:**
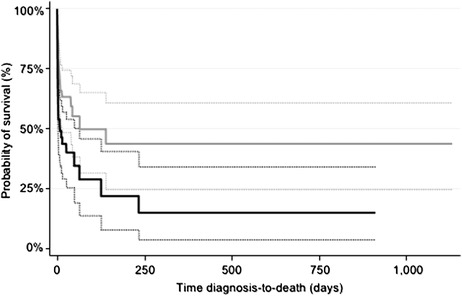
Kaplan‒Meier survival curve and its 95% confidence interval (dashed lines) for canine icterus cases with serum bilirubin concentration at diagnosis ≤60 μmol/L (grey curves) or >60 μmol/L (black curves) sourced from a series of 115 cases diagnosed at two Australian veterinary hospitals between 2015 and 2020.

As revealed by the Kaplan‒Meier survival curve in Figure 2, the hazard of death was not constant, being highest at the time of presentation with a progressively decreasing risk of death over time. For dogs that survived to discharge, the median follow‐up period was 15 days (range: 1–417 days). The MST across all cases was 40 days (95% CI: 9–126 days). The magnitude of serum bilirubin was found to be an important predictor of survival in dogs with hyperbilirubinaemia. Cases with a serum bilirubin greater than 60 μmol/L at initial presentation had a median survival of only 9 days (95% CI: 3–49 days) versus 65 days (95% CI: 15 to >913 days) in cases where serum bilirubin was less than 60 μmol/L at initial presentation. The hazard of death for cases with serum bilirubin >60 μmol/L at presentation was almost double that of cases with serum bilirubin <60 μmol/L (hazard ratio = 1.936; 95% CI: 1.141–3.283), as illustrated by the Kaplan‒Meier survival curve comparison (Figure 3).

## DISCUSSION

This study assessed serum bilirubin concentration in relation to outcome in a population of dogs with a variety of diseases, which were managed medically, surgically or via a combination of the two. We identified a clear difference in survival between dogs with a serum bilirubin above and below a cut‐off of 60 μmol/L, irrespective of the underlying disease process (MST 9 days vs. 65 days, respectively), with an OR of 3.55. This is a higher cut‐off than was reported in a previous study,^13^ where a pre‐operative total bilirubin of 40 μmol/L was significantly associated with non‐survival. Another study reported an association between increasing serum bilirubin concentration and the risk of in‐hospital death in dogs with GBM, with an OR of 1.03. However, using a cut‐off value of 4.3× the upper reference interval, the sensitivity and specificity were poor, at 61% and 63%, respectively.^4^ In contrast, our study showed a clear difference in survival, which may be beneficial in discussions of prognosis with owners and assisting decision making around whether or not to proceed with treatment. Due to the retrospective nature of this study, with relatively small case numbers and 80% study power, interpretation of our cut‐off and application in the clinical setting should be approached with caution.

In the current study, dogs with pre‐hepatic icterus were significantly more likely to die due to their condition than dogs with hepatic or post‐hepatic icterus. Of the 18 dogs diagnosed with pre‐hepatic icterus, 13 were diagnosed with IMHA, four had post‐transfusion haemolysis and one dog had haemolysis secondary to tiger snake envenomation. Of these cases, nine dogs (50%) did not survive to discharge—seven of which had IMHA—and only four dogs were alive at the time of writing, giving an in‐hospital mortality of 53.8% for dogs with IMHA in this study. This is slightly worse than the previously reported guarded prognosis for dogs with IMHA, with the percentage of in‐hospital mortality ranging from 26% to 41%, depending on the study.^6^, ^7^

The overall mortality in this study was approximately 50%, with approximately 35% of dogs failing to survive to discharge. The high mortality rate is not unexpected when we consider the reported in‐hospital mortality rates of 17.4%–68% for GBM,^3^, ^15^, ^16^ 24.5%–27.3% for IMHA,^6^, ^17^, ^18^ 21% for extra‐hepatic biliary duct obstruction secondary to pancreatitis,^19^ with up to 86% in dogs with acute liver failure.^20^ Given that the presence of hyperbilirubinaemia has been shown to be a negative prognostic factor for survival in the majority of these diseases, the high mortality rate in the current study is considered in line with what has been previously reported.

Of all clinical signs observed, pyrexia was the only factor to have a statistically significant impact on outcome, with a decreased risk of death observed in pyrexic dogs (*p*‐value = 0.049). Of dogs presenting with pyrexia, two had pre‐hepatic icterus, ten had hepatic icterus (six of which were diagnosed with infectious cholangiohepatitis) and nine had post‐hepatic icterus (seven of which had EHBDO secondary to pancreatitis). It should be noted that mild pyrexia (or hyperthermia) can occur in dogs which are stressed or in pain. This study defined pyrexia in this study as a rectal temperature above 39.2°C; therefore, it is possible that some dogs were included in this category which were actually hyperthermic rather than truly pyrexic. In this study, the improved survival in pyrexic dogs could be attributable to the presence of diseases from which full recovery is possible, such as ascending cholangiohepatitis, which has a reported survival to discharge of 81.5%,^21^ and EHBDO secondary to pancreatitis, which has a reported survival to discharge of 79%.^19^

This study had a number of limitations due to its retrospective nature. One major limitation was the inconsistency of diagnostic investigation performed, particularly in regard to determining whether the icterus was hepatic in origin. Eleven of the dogs assigned to the hepatic icterus category did not undergo any diagnostic imaging or liver sampling. These cases were assigned based on low clinical suspicion for pre‐ or post‐hepatic icterus based on history, bloodwork, clinical signs and response to treatment. As a result, there may have been cases assigned incorrectly to the hepatic icterus category. Additionally, there is the potential for overlap between icterus types, for example, the possibility of concurrent ascending cholangiohepatitis with GBM, or the presence of hepatic neoplasia with secondary IMHA. Where there was the possibility of overlap, cases were only assigned to one icterus category based on adherence to the criteria described in section ‘Methods’.

A second major limitation is the method used to search the medical records. Cases were included if the medical records contained any of the terms jaundice, icterus or hyperbilirubinaemia; with a serum bilirubin concentration greater than the upper reference interval of 4 μmol/L. As icterus becomes clinically apparent at approximately 35–40 μmol/L, it is therefore possible that some clinicians would not document a marginal bilirubin elevation, thereby missing a subset of lower level hyperbilirubinaemia cases. It should also be noted that a marginal increase in serum bilirubin is not always clinically significant or indicative of serious underlying pathology.

Another limitation is the impact of elective euthanasia on overall survival. It was not always apparent from searching the medical records whether euthanasia was performed due to decline of the condition (and death would have therefore been inevitable) or if financial constraints or personal beliefs of the owners prevented ongoing investigation and treatment. This is a common limitation of veterinary studies and may negatively impact overall survival. It should also be noted that these cases were obtained from two referral hospitals rather than directly from primary accession clinics. Clients presenting to a referral practice may be more financially committed, potentially reducing the number of elective euthanasias as compared with first opinion practice. In addition, cases seen at referral centres may have more complex underlying conditions that require more intensive diagnostic intervention and treatment; they may have poorer prognoses—a factor that is also expected to impact overall survival.

It should be noted that the population of dogs in this study was located in Victoria in southeastern Australia. As such, the prevalence of many infectious diseases considered endemic in many areas worldwide is exceptionally low. Two such diseases with the potential to contribute to hyperbilirubinaemia include babesiosis and leptospirosis, which are rare to non‐existent in the Victorian domestic canine populations. This should be taken into consideration when considering the application of these data to populations of dogs in different geographical locations.

In this study, the prognosis for long‐term survival was poor, with approximately 50% of cases dying or being euthanased as a direct consequence of their condition, with an overall MST across all cases of 40 days from diagnosis to death. Moreover, a serum bilirubin concentration greater than 60 μmol/L at the time of presentation was associated with a significantly shorter MST. This cut‐off may be considered useful in decision making and discussions with owners regarding whether to pursue treatment of dogs presenting with hyperbilirubinaemia. Further prospective studies with larger cohorts may be necessary to establish reliable serum bilirubin cut‐offs to guide prognosis in clinical practice.

## AUTHOR CONTRIBUTIONS

Abigail Brough and Alison Stickney conceived and designed the project. Abigail Brough and Susan Ciaravolo acquired the data. Charles Caraguel analysed and interpreted the data. Abigail Brough and Charles Caraguel wrote the paper.

## CONFLICTS OF INTEREST

All authors declare they have no conflicts of interest.

## FUNDING INFORMATION

The authors received no specific funding for this work.

## ETHICS STATEMENT

The authors confirm that the ethical policies of the journal, as noted on the journal's author guidelines page, have been adhered to. No ethical approval was required as this was a retrospective study. All interventions were deemed clinically necessary and, where relevant, verbal consent was obtained from owners.

## Data Availability

The data that support the findings of this study are available from the corresponding author upon reasonable request.
